# Analysis of Craniocardiac Malformations in *Xenopus* using Optical Coherence Tomography

**DOI:** 10.1038/srep42506

**Published:** 2017-02-14

**Authors:** Engin Deniz, Stephan Jonas, Michael Hooper, John N. Griffin, Michael A. Choma, Mustafa K. Khokha

**Affiliations:** 1 Pediatric Genomics Discovery Program, Department of Pediatrics, Yale University, 333 Cedar Street, New Haven, CT 06510, USA; 2 Department of Medical Informatics, RWTH Aachen University, Pauwelsstraße 30, 52074 Aachen, Germany; 3 Department of Radiology and Biomedical Imaging, Department of Pediatrics, Yale University, 333 Cedar, Street New Haven, CT 06510, USA; 4 Department of Biomedical Engineering and Applied Physics, Yale University, 333 Cedar Street, New Haven, CT 06510, USA; 5 Department of Genetics, Yale University School of Medicine, 333 Cedar Street, New Haven, CT 06510, USA

## Abstract

Birth defects affect 3% of children in the United States. Among the birth defects, congenital heart disease and craniofacial malformations are major causes of mortality and morbidity. Unfortunately, the genetic mechanisms underlying craniocardiac malformations remain largely uncharacterized. To address this, human genomic studies are identifying sequence variations in patients, resulting in numerous candidate genes. However, the molecular mechanisms of pathogenesis for most candidate genes are unknown. Therefore, there is a need for functional analyses in rapid and efficient animal models of human disease. Here, we coupled the frog *Xenopus tropicalis* with Optical Coherence Tomography (OCT) to create a fast and efficient system for testing craniocardiac candidate genes. OCT can image cross-sections of microscopic structures *in vivo* at resolutions approaching histology. Here, we identify optimal OCT imaging planes to visualize and quantitate *Xenopus* heart and facial structures establishing normative data. Next we evaluate known human congenital heart diseases: cardiomyopathy and heterotaxy. Finally, we examine craniofacial defects by a known human teratogen, cyclopamine. We recapitulate human phenotypes readily and quantify the functional and structural defects. Using this approach, we can quickly test human craniocardiac candidate genes for phenocopy as a critical first step towards understanding disease mechanisms of the candidate genes.

Birth defects affect 3% of children in the United States (Centers for Disease and Prevention, 2008) and are the most common cause of infant death^1^,^2^,^3^. Among the birth defects, Congenital Heart Disease (CHD) is the most common^2^,^4^, affecting 1.3 million newborns per year worldwide. Furthermore, in patients with CHD, the most frequent non-cardiac anomaly is skeletal defects (35%) followed by gastrointestinal (25.2%), renal (23.1%) and craniofacial defects (19.7%)^5^, reflecting intersecting pathways during development that are not well explored. Importantly, the high prevalence of craniofacial defects in CHD may reflect the critical role of the neural crest in both craniofacial and cardiac development (craniocardiac). The neural crest is a multipotent cell population that forms at the borders of the dorsal neural tube and eventually delaminates and migrates throughout the embryo. Neural crest derivatives play a role in both cranial and cardiac development^6^,^7^,^8^, providing at least one relation between both craniofacial and cardiac development. However, our understanding of the molecular pathways that pertain to craniocardiac development remain incomplete.

In order to define the genetic causes of CHDs, including craniocardiac malformations, the NIH/NHLBI established the Pediatric Cardiac Genomics Consortium (PCGC) under the Bench to Bassinet program^9^. The PCGC seeks to discover genes that may cause CHD. Unfortunately, due to high locus heterogeneity, only a small percentage of these candidate genes have second unrelated alleles to validate them as disease causing^10^,^11^. In addition, many of the variants have no known role in craniocardiac development, no known role in embryonic development in general, or in other cases, any known function. Therefore, there is a pressing need to use rapid animal models for functional screening prior to mechanistic studies.

For functional screening of craniocardiac candidate genes, we propose to model these diseases in *Xenopus tropicalis*, an inexpensive and rapid amphibian model, where cardiac and facial structures can be simultaneously assessed. *Xenopus* is readily manipulated either by gain of function by simply injecting mRNAs into the fertilized egg or loss of function via ‘antisense oligos‘ (morpholinos) that deplete gene products either by preventing mRNA translation or splicing. In addition, CRISPr/CAS9 gene editing technology works well in *Xenopus*^12^,^13^ including rapid modeling of disease phenotypes in F0 embryos^14^. There are now well over 350 CHD candidate genes, and therefore, a rapid, inexpensive model system is essential^10^,^11^,^15^. Mammalian model systems are simply too expensive and too slow to screen this number of candidate genes. *Xenopus*, on the other hand, is the most closely related model to humans that retains a high degree of manipulability and low cost. In addition, most high-resolution phenotyping methods such as histology are also prohibitive due to the labor and time required. Instead, optical coherence tomography (OCT) can discern internal structures efficiently.

Analogous to clinical ultrasonography, OCT provides cross-sectional imaging of tissues but employs light rather than sound waves^16^. OCT uses multiple longitudinal scans to gather structural information from reflected and backscattered light. OCT provides high-speed, cross-sectional, label free imaging, which can outline dynamic *in vivo* morphology and function at micrometer resolution. Typically, axial and lateral resolutions range between ~3–10 μm and ~5–20 μm respectively which approaches the resolution of histology. Image acquisition produces movies, which can discern dynamic tissue structures like the heart. Importantly, tissue preparation is trivial eliminating the need for fixation, excision, labeling and processing^17^. OCT has become a standard imaging tool for clinical diagnoses of retinal diseases in ophthalmology^18^. Besides these clinical applications, OCT has demonstrated effectiveness in the developing heart of embryonic models including *Drosophila*^19^,^20^,^21^, avian^22^,^23^,^24^,^25^,^26^,^27^, mouse^28^,^29^,^30^,^31^,^32^, zebrafish^33^,^34^,^35^ and *Xenopus*^36^,^37^,^38^,^39^. Recently, researchers modeled fetal alcohol syndrome in the avian model, where the human teratogen, ethanol, creates ventricular septal defects as well as misaligned vessels. OCT readily resolved these cardiac defects in the developing avian embryo^26^. In zebrafish, OCT can resolve the two-chambered heart structures dynamically with *in vivo,* live imaging^33^,^40^. Finally in Xenopus laevis, Doppler OCT has demonstrated utility in measuring cardiac function^37^.

Although the efficacy of OCT in *Xenopus* as well as other models is established, we currently lack a rapid, efficient and reproducible approach to imaging cardiac and facial structures in high-throughput model systems that would be amenable to candidate gene screening for craniocardiac malformations. In addition, we have not yet defined normative values for these structures nor have we demonstrated that these parameters are altered in modeled diseases. To address this problem and capitalize on the benefits of OCT applied to *Xenopus* tadpole morphometry, we first need to establish imaging guidelines and reference data similar to clinical ultrasonography. This will provide a foundation to interrogate and quantify craniocardiac structures during normal development and when modeling disease states.

In this study, we sought to image *Xenopus tropicalis* craniocardiac structures in order to establish normative measurements of anatomy, establish standard protocols to measure these structures quantitatively, and then image disease phenotypes in order to detect even subtle alterations in anatomy and function during early stages of development. By phenocopying patient’s phenotypes in *Xenopus*, we bolster the evidence that the candidate gene may be disease causing. Our fast and efficient approach aims to improve candidate “genes-to-functions screens” that can act as a springboard for mechanistic studies of craniocardiac malformation genes.

## Results

### *Xenopus* Cardiac Imaging and Quantitation

To diagnose CHD in clinical cardiology, two-dimensional (2D) transthoracic echocardiography is the most common imaging modality used. In order to understand three-dimensional morphology and function, the clinician images multiple planes in a consistent and reproducible manner that enables quantification. Our overall goal is to establish *Xenopus* as a rapid throughput model for screening CHD and craniofacial malformation candidate genes. Therefore, we sought to develop a similar imaging principle using *Xenopus* and OCT.

**Figure 1:**
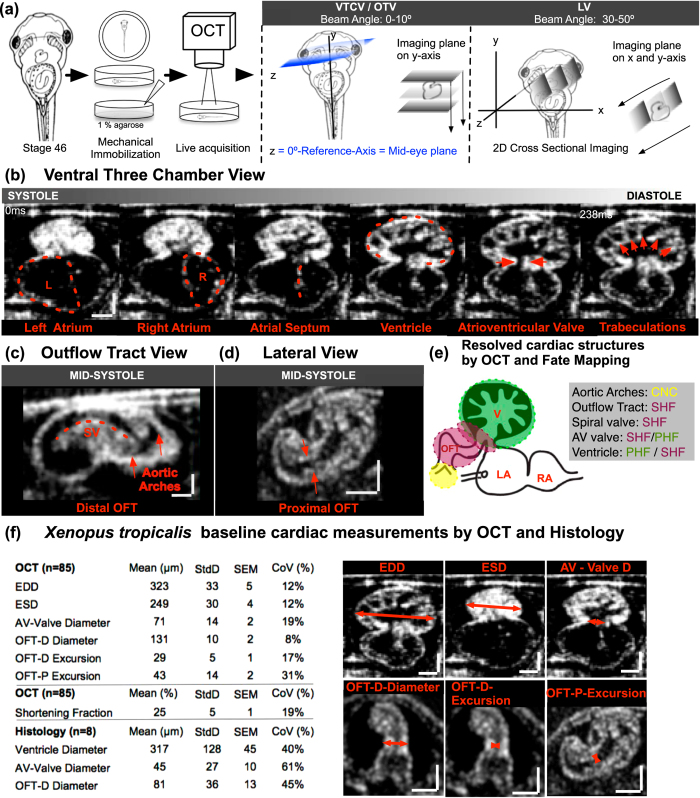
Imaging the *Xenopus tropicalis* heart with OCT. (**a**) Stage 46 tadpole embedded in 1% agarose and positioned so that the ventral side faces the OCT lens. Next, using a 5-axis stage, the embryo is oriented to the reference axis-mid eye plane. Finally, the heart is scanned from various angles to capture the three imaging planes. To capture the Ventral Three Chamber View (VTCV) and Outflow Tract View (OTV) we set the OCT imaging plane ± 0–15° to the reference axis and manually move the embryo posteriorly towards the tail on the y-axis until we capture the ventricle and the outflow tract. **Similarly, to capture the Lateral View (LV) we adjusted the imaging plane** ± 30–50° to the reference axis (**b**) **Ventral Three Chamber View**: Visualizes the single ventricle and both atria. Red dashed line marks the left atrium, right atrium, atrial septum, ventricle, atrioventricular valve, and trabecula in different stages of the cardiac cycle from systole to diastole (left to right). **(c) Outflow Tract View**: By moving the imaging plane anteriorly to the cranium, OCT captures the distal outflow tract including the spiral valve and systemic arteries. **(d) Lateral View:** Rotating the OCT beam angle 30–50 °C reveals the proximal outflow tract and the ventricle visualized during mid-systole. **(e) Cardiac structures Resolved by OCT and Fate Mapping**: Aortic arches; outflow tract, spiral valve and atrioventricular valve and ventricle; ventricle populated by primary and secondary heart field. (**f**) *Xenopus tropicalis* wildtype cardiac measurements by OCT and Histology. EDD: end diastolic diameter, ESD: end systolic diameter AV: atrioventricular, OFT: outflow tract, P: proximal, D: distal, StdD: standard deviation, SEM: Standard error of the mean, CoV: Coefficient of variation. Y-X Scale bar: 100 μm.

Clinical echocardiography begins with imaging in specific planes including: parasternal, apical, subcostal and suprasternal planes. Each plane focuses on a particular cardiac structure and establishes a reference to compare malformations. To begin our studies, we embedded stage 44–46 (post fertilization day 3 raised at 28 °C) tadpoles in an agarose gel to position the ventral cardiac sac towards the OCT beam for imaging (Fig. 1a). Importantly, we refrained from employing paralytic agents (such as benzocaine) as we noted alterations in cardiac function with paralysis. We first defined a zero-degree reference axis where a plane intersecting the tadpole’s eyes and the tip of the tail is orthogonal to the imaging beam (Fig. 1a). This position served as a starting point for imaging and all the following imaging planes are defined with respect to this reference plane. Next, we defined three planes modeled on clinical echocardiographic imaging planes (Fig. 1 and Supplementary Fig. 1):

### Ventral Three-Chamber View (VTCV)

The VTCV resembles the ‘Apical Four Chamber View’ of clinical echocardiography where all four chambers and both tricuspid and mitral valves are visualized in humans. Similarly, In *Xenopus,* the VTCV visualizes the left atrium, right atrium, incomplete atrial septum, ventricle, ventricular trabeculations and the atrioventricular valve (AV-valve) (Fig. 1b). To capture the VTCV, we set the OCT imaging plane ± 0–15° to the reference axis and manually move the embryo posteriorly towards the tail on the y-axis until we capture the ventricle. In this view, we quantitate two features:

1. AV-Valve: Once the ventricle is captured on the y-axis, the stage is adjusted on the z-axis to capture the AV-valve. This is a useful view to quantify maximal AV-valve leaflet separation (which we will refer to as valve ‘excursion‘ distance) and to analyze valve morphology and compliance.
2. Ventricle: The ventricle is the most ventral cardiac structure placing it most adjacent to the OCT system. In the VTCV, we can visualize the highly trabeculated myocardium which contracts longitudinally, starting from the apex toward the valves. We can analyze the depth of trabeculation, wall thickness and dynamics of the ventricular contraction (Fig. 1b). We can estimate a shortening fraction based on the ventricular systolic and diastolic diameters, which we use as a surrogate for ventricular function similar to clinical echocardiography.

### Outflow Tract View (OTV)

The OTV is similar to the ‘Suprasternal view’ in clinical echocardiography, which is commonly used to examine the great vessels. In *Xenopus,* we obtain this view by adjusting the imaging plane ± 0–10° to the reference axis similar to the VCTV view but move the embryo anteriorly on the y-axis towards the cranium. The OTV visualizes the truncus arteriosus, spiral valve and the distal systemic vessels (Fig. 1c). The truncus arteriosus conducts the outflow from the ventricle and contains the spiral valve, which initiates from the proximal end and spirals within the truncus to the distal end. During the ventricular systole, the spiral valve splits the blood flow within the truncus. As the cardiac cycle continues into ventricular diastole, the spiral valve creates a tight seal to prevent regurgitant flow from the systemic arches. The truncus then divides into the right - left distal arteries and progresses to the systemic and pulmunocutaneous arches, respectively. All these structures can be resolved by OCT in real time in the OTV (Fig. 1c).

### Lateral View (LV)

The LV is analogous to the ‘parasternal long axis view’ of clinical echocardiography where the ventricles, mitral valve, aortic root and aortic valve can be assessed. By adjusting the OCT imaging plane to 40–50° from the reference axis and moving the embryo along the y and x-axis posteriorly towards the tail until the proximal outflow tract is as horizontal as possible to the imaging plane, we can visualize most of the proximal outflow tract as well as the ventricle in *Xenopus* (Fig. 1d). In this view, we measure the maximal proximal spiral valve leaflet separation (we will again refer to leaf separation as ‘excursion‘) to assess OFT dynamics (Fig. 1f). Combining the LV and the OTV, we can assess the truncus in two parts. The LV focuses on the proximal truncus, while the OTV focuses primarily on the distal truncus. These measures are useful since the proximal and the distal outflow tract have different embryonic origins, the secondary heart field and the cardiac neural crest respectively^41^ (Fig. 1e). Therefore, malformations confined to either the distal or the proximal part of the OFT can be delineated by OCT.

A number of other cardiac parameters can be measured including the maximal end diastolic diameter (EDD), maximal end systolic diameter (ESD), maximal atrioventricular valve excursion, outflow tract distal-end diameter, outflow tract distal-end excursion and outflow tract proximal-end excursion. We use ‘diameter‘ to define the wall-to-wall distance and ‘excursion‘ to define the maximal separation of leaflets of the AV-Valve and the Outflow tract during atrial and ventricular mid-systole respectively (Fig. 1f and Supplementary Fig. 1).

### Cardiomyopathy in *Xenopus* can be phenotyped by OCT imaging

Having established OCT imaging as a rapid method for analyzing *Xenopus* cardiac anatomy, we next sought to examine well-established human disease states. To model human cardiomyopathy^42^, we examined myosin heavy chain 6 (MYH6) which disrupts the sarcomere, the contractile unit of the myocardium. In addition, a forward genetic screen in *Xenopus* identified a *myh6* mutation as a cause for non-contractile hearts with severely disrupted cardiomyocyte cytoskeleton^43^. We knocked down *myh6* with antisense morpholino oligonucleotides (MO). We raised morphants (MO injected tadpoles) to stage 44–46 and applied OCT using our established imaging planes. At low doses of MO (0.5 ng), morphants did not show any abnormalities whereas at high doses of MO (2–4 ng) tadpoles were severely malformed and did not survive to cardiac imaging stages (data not shown). To test if our OCT system could detect subtle phenotypes, we titrated the dose of MO to 1 ng so that morphants had normal overall gross morphology with no visible cardiac abnormalities under simple stereomicroscopy.

**Figure 2:**
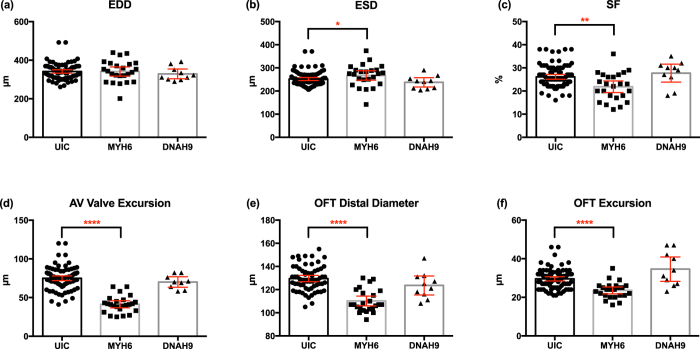
Quantitative assessment of the tadpole hearts using OCT with cardiomyopathy and heterotaxy. Cardiomyopathy model generated by myosin heavy chain 6 (MYH6) knockdown and heterotaxy model generated by dynein heavy chain 9 knockdown (DNAH9). Measurement of **(a)** EDD, **(b)** ESD **(c)** SF **(d)** AV valve excursion distance **(e)** OFT wall-to-diameter **(f)** OFT excursion distance. UIC: uninjected control, EDD: end diastolic diameter, ESD: end systolic diameter, OFT: outflow tract, SF: shortening fraction. (Mann-Whitney test; p < 0.05) (*p < 0.05/**p < 0.01/****p < 0.0001) (Bars represent mean with 95% confidence interval).

In the VTCV, we measured the maximal End Diastolic Ventricle Diameter (EDD), End Systolic Ventricle Diameter (ESD) and the Atrio-ventricular Excursion Distance during the left atrial contraction (Fig. 2). From these data, we can also derive an addition parameter: percent change in the total ventricular diameter which we define as shortening fraction (*Shortening Fraction; SF* = (*EDD*-*ESD*)/*EDD* * 100), which can be used to assess pump function. Analyzing these measurements (Fig. 2), *myh6* morphants had similar EDD but mildly increased ESD when compared to the uninjected controls. Morphant hearts had slightly higher end systolic diameters suggesting systolic dysfunction. The shortening fraction was significantly less in *myh6* morphants compared to uninjected controls. However, other cardiac structural parameters were unchanged. These findings suggested preserved myocardial architecture but altered pump function, as expected, due to the reduction in myosin. Next we examined the AV-valve. Morphant hearts had a significantly lower AV-valve excursion distance than uninjected controls suggesting either poor pump function or a non-compliant valve. Combining a reduced SF, smaller AV-Valve excursion, and dilated atria in the lateral view, these findings suggest that a non-compliant, poorly contracting ventricle leads to decreased cardiac output (reduced AV valve excursion) and dilated atria.

We then focused on two parameters in the OTV; Distal outflow tract maximal excursion distance and distal outflow tract maximal wall to wall diameter. The excursion distance is best measured at the ventricular mid-systole when the outflow tract is at maximal dilation, at this point spiral valve leaflet separation is at the maximal point. Then we repeated the measurements during ventricular diastole when the outflow tract is empty. In *myh6* morphant hearts this distance was significantly reduced compared to wild-type controls (Fig. 2e). The *myh6* morphant outflow tract was much narrower than the wild-type controls and had a smaller excursion distance.

Finally, in the LV, we visualized the proximal outflow tract where the spiral valve initiates. However, we did not quantify the OFT-P due to high CoV levels in controls.

Next we sought to determine the robustness of our assay in the face of a malpositioned hearts. For example, in humans, loss of cilia motility can lead to *situs inversus*, which leads to a mirror arrangement in cardiac *situs* but typically does not cause any other alteration in cardiac structure or function. In this situation, other than a *situs* change, clinical echocardiography does not reveal any abnormalities. We asked if our model system would perform similarly and recapitulate the human phenotype. We depleted *dnah9 (dynein heavy chain 9*) which is an established human ciliopathy gene that affects cilia motility and leads to situs inversus^44^,^45^,^46^,^47^. We used the CRISPR/Cas9 system to deplete *dnah9*^14^ and identified 10 tadpoles with reversed cardiac looping by visual inspection under stereomicroscopy and compared them to UICs as well as the *myh6* morphants. Despite the malposition of the heart in *dnah9* morphants, we found no difference in our measurements of cardiac parameters (Fig. 2).

In summary, here we show by OCT imaging that we are able to delineate structural and functional abnormalities such as cardiomyopathy in tadpole hearts quickly which cannot be quantitated effectively under stereomicroscopy. Furthermore, we verified that our measurements were not affected by gross heart malpositioning that otherwise does not affect internal cardiac anatomy. Here we conclude that our approach can be an effective tool to screen human candidate genes for congenital heart disease.

### OCT can rapidly resolve craniofacial cartilage structure

Next, given the co-incidence of craniofacial malformations with cardiac malformations, we wondered if OCT could also readily visualize and quantitate craniofacial elements. The craniofacial structures are complex and difficult to resolve by simple stereomicroscopy even when various lighting methods such as dark field illumination are employed. Therefore, we hoped that with minimal additional effort, OCT could provide detailed information about the craniofacial microanatomy. In fact, because the craniofacial anatomy is static unlike the dynamic cardiovascular structures, we could delineate craniofacial structures at very high resolution without any additional processing of the embryo.

**Figure 3:**
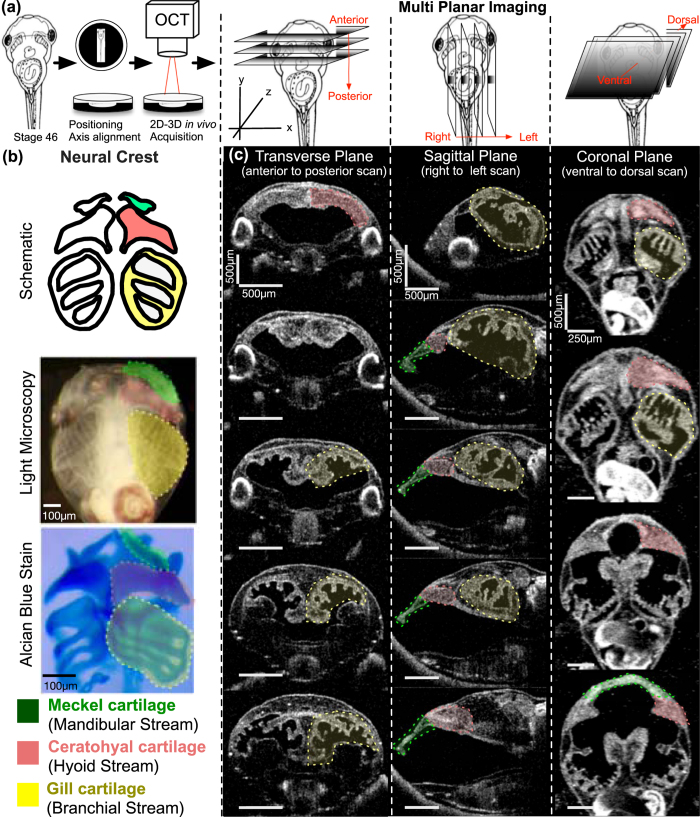
Imaging *Xenopus* craniofacial structures with OCT **(a)** We create a slit at the center of the clay with the Stage 46 tadpole positioned ventrally towards the OCT imaging plane (XZ-plane). Then we adjust the 5-axis stage to capture a zero- degree reference axis where a plane intersects the tadpole’s eyes symmetrically and the tip of the tail. This plane defines the 0° orthogonal plane. We then move this imaging plane in y, x and z axes to capture transverse, sagittal and coronal sections respectively. Imaging planes are adjusted on all three axes to capture distinct facial structures. **(b)** Top panel shows the schematic representation of the three neural crest streams: mandibular (green), hyoid (pink) and branchial (yellow) which form Meckel, ceratohyal and gill cartilages respectively. Each structure is highlighted under simple stereomicroscopy image (middle panel) and after alcian blue stain (bottom panel) (**c)** OCT images of craniofacial structures in all three planes: Transverse, Sagittal and Coronal. Meckel’s cartilage (green label) is best viewed in sagittal (yz-axis) and coronal (xy-axis) sections. The ceratohyal cartilage can be easily resolved by the coronal sections. The gill cartilages are more posterior compared to Meckel’s and ceratohyal cartilage, and the gill cartilages occupies most of the craniofacial space. Qualitative analyses can be made in all axes.

In order to systematically quantitate craniofacial structures, we chose to organize our analysis based on embryological origin. In particular, the neural crest is essential for the formation of craniofacial structures^48^. At the border of the neural tube, the neural crest is specified and then migrates to a variety of locations including the heart and craniofacial structures. During the formation of the craniofacial structures, the cranial neural crest cells migrate into the face as three distinct streams. Each stream invades particular pharyngeal arches that go on to form specific facial structures including the craniofacial skeleton, a highly conserved process across the vertebrates including humans. We can then divide our analysis of craniofacial structures based on their embryological origin from these neural crest streams: Mandibular, Hyoid, and Branchial Streams (Fig. 3b). Here, we show that OCT can delineate specific cartilaginous structures originating from each stream, enabling the rapid assessment of facial and cardiac development with resolutions close to histology (Supplemental Fig. 2). As in cardiac imaging, we define specific optimal planes, which are necessary for quantitation.

Similar to our *Xenopus* cardiac imaging strategy, which we modeled on clinical echocardiography, here we modeled craniofacial structure imaging to computerized axial tomography (CT) imaging. Following a full cardiac exam by OCT, the tadpole is extracted from the agarose with multiple 1/9xMR washes and transferred to a new petri dish coated with black, oil based clay with a 9 mm × 9 mm × 3 mm well manually situated in the center. We create a slit at the center of the well using watchmaker forceps (length ~4–5 mm) in which the tadpole is situated ventrally (Fig. 3a). Then we adjust the 5-axis stage to capture a zero- degree reference axis where a plane intersects the tadpole’s eyes symmetrically and the tip of the tail. This plane defines the 0° orthogonal plane. We then move this imaging plane in y, x and z axes to capture transverse, sagittal and coronal sections respectively (Fig. 3c). For qualitative assessment, we scanned the craniofacial structures from most anterior tip of the tadpole to the cardiac sac in each of the x, y, and z-axes similar to CT scanning in order to visualize the facial structures at various angles. Then we identified optimal planes for qualitative assessment of each cartilage element. Below, we divide this analysis based on the neural crest streams:

#### Mandibular Stream

The mandibular stream develops into Meckel’s cartilage^49^,^50^,^51^ which forms most of the anterior part of the lower jaw (Fig. 3b). Meckel’s cartilage is best viewed in sagittal (yz-axis) and coronal (xy-axis) sections (Fig. 3c). The sagittal view was best for assessing its relation to the ceratohyal cartilage. For quantitative analysis, we utilized the coronal sections, moving the stage along the z-axis until we capture the most anterior tip of Meckel’s cartilage as well as the most posterior end, which eventually forms the ceratohyal articulation. Once this plane is captured, we measure a line between these two points traveling along the mid-corpus of the cartilage (Fig. 5b).

#### Hyoid Stream

The hyoid stream gives rise to the **ceratohyal cartilage**^51^, which can be easily resolved by the coronal sections on the xy-axis (Fig. 3b and c). This triangular cartilage thins out as it extends to the lateral aspect of the cranium. On coronal sections, we adjust the plane (by tilt/yaw ± 4°) to discern the longest ceratohyal axis, extending from the midline to the most lateral portion of the cartilage. We measure the length along the corpus from this midline to lateral margin (see below). Another helpful plane is the sagittal section on the xz-axis which allows for a qualitative assessment of the ceratohyal cartilage thickness and relation to Meckel’s cartilage (Fig. 3c).

#### Branchial Stream

The branchial streams give rise to the gill cartilages^51^, which in exclusively aquatic tadpoles are essential for oxygen exchange (Fig. 3b). Interestingly in OCT but not by stereomicroscopy, we can readily visualize villi in the gills that are well vascularized presumably to enhance surface area for gas exchange. Interestingly, these structures are difficult to visualize by histology possibly since some of the architecture changes with fixation, dehydration, and wax embedding. The gill cartilages are more posterior compared to Meckel’s cartilage, and the gill cartilages occupies most of the craniofacial space. We can make qualitative analyses in all axes. For quantitation, we made measurements in the coronal sections on the xy- axis (Fig. 3c). We positioned the tadpole on the ventral side and scroll along the z-axis until the largest mid-lateral portion of the gill cartilages is visualized and acquire a set of 3D data by OCT. We then draw two points between the largest middle and lateral borders along the corpus of the most anterior gill.

In order to establish an efficient screening procedure, we defined normative values only on coronal sections (XY Axis) where all three streams can be visualized on the same plane to minimize processing time. We also limited measurements to one side of the tadpole to further utilize the advantage of *Xenopus* lineage. By injecting one cell at the two-cell stage, an experimenter can select embryos where the manipulated side is either on the left or right of the tadpole with the other side unaffected and useful as an internal control. Therefore, an uninjected control side and the perturbed half can be compared easily^52^. Our goal is to develop a fast, high-throughput and efficient system, therefore we seek to optimize our measurements to minimize processing time.

### OCT imaging detects neural crest defects in *Xenopus*

**Figure 4:**
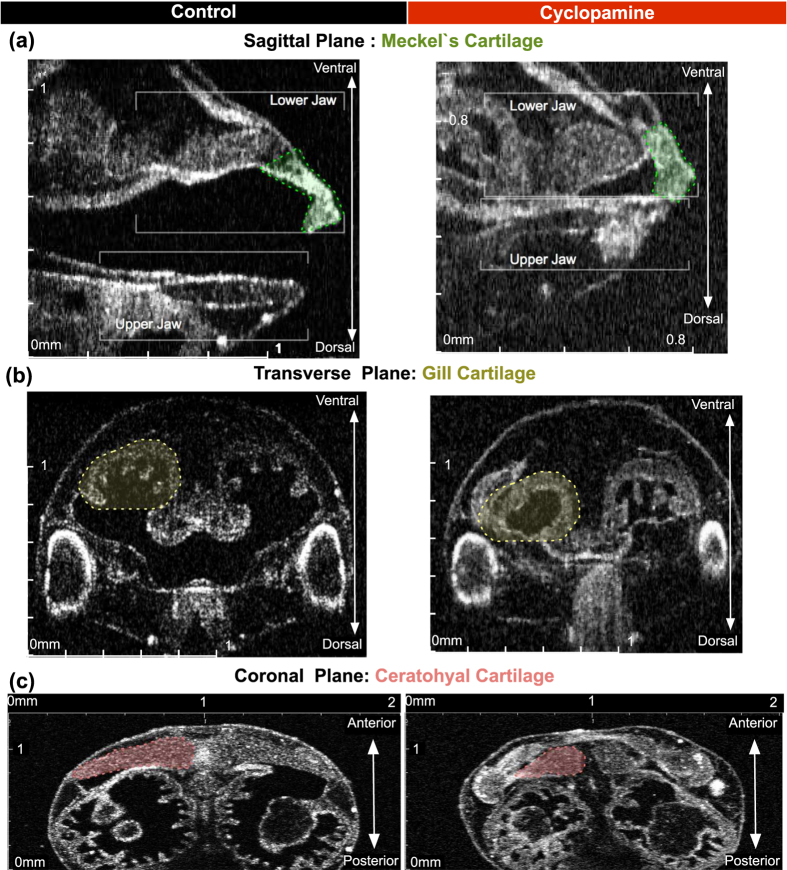
Qualitative neural crest defects in *Xenopus* with cyclopamine treatment. Stage 46 uninjected control tadpole compared to cyclopamine treated tadpoles and demonstrated the teratogenic effects. **(a–c)** Left column shows the control and the right column shows the cyclopamine treated tadpole. In cyclopamine treated tadpoles both jaws were short and thick. Meckel and ceratohyal cartilage were smaller and gill cartilages were nearly lost. (Scale Bar on x and y axis: 0–2 mm).

**Figure 5:**
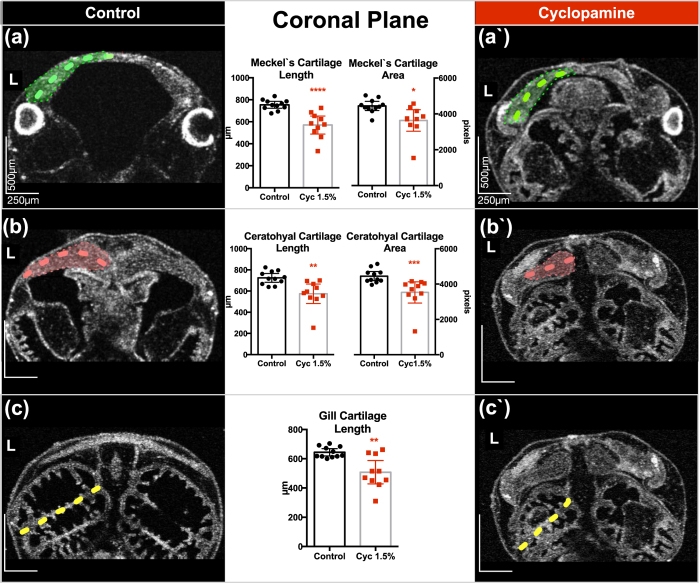
Quantitative neural crest defects in *Xenopus* with cyclopamine treatment. **(a/a′–c/c′)** Stage 46 control tadpole compared to cyclopamine (2.5 mg) treated tadpoles. The tadpole is positioned on the ventral side up, and the OCT imaging plane scrolled along the z-axis until the largest mid-lateral portion of the cartilages is visualized. We acquired a set of 3D data. On these coronal sections a line between two points traveling along the mid-corpus of the Meckel’s and ceratohyal cartilage is measured. Then the largest middle and lateral borders along the corpus of the most anterior gill are marked, and the midline length is measured. Quantitative analysis demonstrated reduction in length and area in treated tadpoles on the coronal plane. L: left, (Mann-Whitney test; p < 0.05) (*p < 0.05/**p < 0.01/****p < 0.0001) (Bars represent mean with 95% confidence interval).

Cyclopamine is a steroid alkaloid teratogen that causes fatal human birth defects, holoprosencephaly and cyclopia. It inhibits the hedgehog-signaling pathway by directly binding to its receptor Smoothened. In *Xenopus*, hedgehog signaling is required for the formation, maintenance and migration of the neural crest^53^ and cyclopamine causes craniofacial defects. Here we use cyclopamine to perturb cartilage formation and demonstrate that OCT can delineate subtle defects in different craniofacial elements. We treated tadpoles with 2.5 μM of cyclopamine from stage 14 to stage 28. The dose is titrated down to a minimal level where under stereomicroscopy no obvious craniofacial defect was detected (Figs 4 and 5).

#### Sagittal plane- YZ Axis

This view resolved both upper and lower jaw formation as well as the oral cavity, which was significantly smaller and nearly non-existent in cyclopamine treated tadpoles. Both jaws were shorter and thicker when compared to untreated controls (Fig. 4a).

#### Transverse Plane- XZ-Axis

In this view, we can assess the gills as they project into the oral cavity. In cyclopamine treated tadpoles, the gills were nearly completely lost (Fig. 4b).

#### Coronal Plane- XY-Axis

In this view, we can readily assess the ceratohyal cartilage as well as quantify all three cartilages (Figs 4c and 5). We measured Meckel’s cartilage maximal length/area, the ceratohyal cartilage maximal length/area, and finally the gill cartilage’s maximal length. We confined these measurements to the left side of the tadpole in order to streamline the process.

Cyclopamine treated tadpoles, as expected, demonstrated shorter and smaller cartilage when compared to untreated controls (Fig. 5).

## Discussion

Ongoing human genetic studies are identifying sequence variations in CHD subjects at a rapid rate^54^, but functional testing of these candidate genes is sorely lacking. There is a pressing need to develop rapid model systems for testing these candidates since these patients have high locus heterogeneity, which makes it difficult to conclude whether candidates are really causative for CHD. In addition, so many of the candidate genes are novel to cardiac development, functional testing is essential to begin to dissect pathogenic mechanisms. Here we show that the high-throughput, inexpensive model system *Xenopus* can be paired with OCT imaging to investigate the direct functional impact of CHD genes on craniocardiac development. For example, once a candidate sequence is identified, loss of function can be modeled using morpholinos and/or CRISPR/CAS9 system in less than 1–2 weeks. Importantly, with CRISPR based F0 gene modification in *Xenopus*, the cost of gene depletion is 10 fold less expensive than MOs enabling screens of hundreds of candidate genes^14^. With our standard OCT imaging protocol, we can acquire the necessary raw data in 10–15 min per tadpole, with complete image processing and analysis by the next day. We can acquire complete cardiac and facial data for ~50 tadpoles per day (9 hour day). Therefore, for craniocardiac development a candidate gene can be comprehensively analyzed using *Xenopus* and OCT within 1 week. Depending on the experimental setup, if only cardiac or only cranial phenotyping is planned, image acquisition can be reduced to ~5 min, enabling ~100 tadpoles to be imaged per day (9 hours day). This is considerably faster and dramatically less expensive than mammalian model systems. Furthermore, with the fecundity of Xenopus and this short acquisition rate, we can acquire quantitative emeasurements on a large number of controls as well as manipulated embryos, enabling statistical evaluation and controlling for variations in a clutch. The *Xenopus* cardiovascular system develops within 72 hours and remains optically accessible throughout these early stages of development^55^. Most importantly, oxygen delivery in tadpoles relies on simple diffusion rather than cardiac output. Even in the presence of a non-beating heart, tadpoles survive for several days, which is a major advantage when studying severely disrupted cardiogenesis in some forms of CHDs. For example, in the mouse, cardiac function is essential and impairment leads to early embryonic lethality making the analysis more difficult.

When compared to fish models, there is a high degree of evolutionary conservation between *Xenopus* and humans as evidenced by the large degree of synteny present between frog and human genomes that is lost when compared to fish^56^. The zebrafish heart is two chambered, atrium and ventricle, while the *Xenopus* heart is three-chambered, two atria, atrial septum, a highly trabeculated single ventricle, atrioventricular valve, and an outflow tract resembling higher vertebrates more closely. Finally, the genome of *X. tropicalis* has not undergone genome duplication like teleost fish or *X. laevis* making it more amenable to gene knockdown by morpholino oligonucleotides (MO) since fewer alleles need be targeted. MO are antisense oligos that are targeted to either the translation start site of an mRNA or splice site of pre-mRNA inhibiting translation or splicing respectively and culminating in protein knockdown. It is trivial to inject hundreds of embryos with different doses of MOs in a single sitting. One substantial advantage of MOs is that gene dosage can be titrated based on amount of MO injected providing fine dosage control. The genetic analysis of most CHD patients to date has focused on *de novo* mutations, which are heterozygous suggesting that gene dosage is critical for disease pathogenesis^15^. Compared to mouse knockouts, MO knockdown is drastically less costly and much more rapid, therefore, achieving an important balance between cost and human evolutionary conservation. We favor a model where *Xenopus* modeling identifies strong candidates that can then be additionally tested in mouse for mammalian specific phenotypes.

Given the large number of candidate genes already identified in CHD and the expectation for many more, histology can be quite time consuming making these strategies infeasible for screening. Moreover, a large spectrum of disease phenotypes are simply too difficult to detect effectively with simple stereomicroscopy. OCT alleviates many of these challenges by optimizing sensitivity and efficiency. Importantly, OCT imaging is non-destructive. Therefore, we can trace a disease process as it develops and visualize its progression during development in the same set of embryos. For example, cardiac structures are serially connected contractile structures. Any malformation can lead to stenosis, regurgitation, or hypoplasia leading to dramatic changes in the intracardiac biomechanical forces. Therefore, the primary defect can cause a cascade of secondary defects due to these changes. By following development over time using OCT, primary defects can be easily distinguished from secondary effects. For this reason, before applying mechanistic studies, it is crucial to map these structural defects at the earliest time point and visualize progression during the disease state in order to identify the primary defect. Therefore, the non-destructive nature of OCT imaging allows us to monitor and image the same embryo over many time points to further observe heart malformations over time, adding another layer to the strength of the model for screening. These phenotypes can be easily missed with histological assessments at one time point.

Our OCT setup has axial resolution of less than 7.5 μm and a lateral resolution of 15 μm in air (4.9 μm in water) enabling the imaging of stage 44–46 tadpoles, which has been our primary focus. However, the heart tube forms at stage 31–33 (NF Stages), contractions begin at stage 35, and cardiac looping takes place around 33–36. The valves and atrial septum form at stage 44–45 (Lohr and Yost, 2000). As the field continues to advance and ultra-high-resolution 3D-OCT technologies (axial resolution 1–3 μm) are becoming more available earlier steps of cardiogenesis as well as facial development can be further visualized. Future work will focus on delineating these structures at these earlier stages.

We were delighted to see that craniofacial imaging in *Xenopus* can be so revealing without much additional effort. Craniofacial malformations are a major additional morbidity to congenital heart disease and so the addition of this imaging allows us to increase our disease modeling and functional testing. In addition, by delineating a craniofacial phenotype, we can examine shared embryological origins for defects improving our ability to understand the pathogenic mechanisms. Together, the use of human disease gene discovery with massively parallel sequencing technologies and OCT imaging in our rapid disease model *Xenopus* has the potential to transform our understanding of craniocardiac development and the causes of these terrible diseases of infancy.

## Materials and Methods

### *Xenopus* husbandry

*Xenopus tropicalis* were housed and cared for in our aquatics facility according to established protocols that were approved by the Yale Institutional Animal Care and Use Committee (IACUC).

### Microinjections

*In vitro* fertilization and microinjections were done as previously described (del Viso and Khokha, 2012) and protocols are available on our website (http:// http://khokha.medicine.yale.edu/). Embryos were injected at the one cell stage. For microinjections, borosilicate glass needles were calibrated to inject 1 ng MYH6 morpholino oligonucleotide (5′-AGTCTGCCATCAGGGCATCACCCAT-3′, Gene-Tools, LLC) with tracer Alexa 488 (Invitrogen). CRISPR sgRNAs were designed for DNAH9 as previously described and injected with Cas9 protein^14^. After injections, embryos were left in 1/9X MR + 3% Ficoll for 1–2 hr and then transferred to 1/9X MR supplemented with 50 μg/ml of gentamycin. Injections were confirmed by fluorescent lineage tracing with a Zeiss Lumar fluorescence stereomicroscope at stage 28–30 and tadpoles further incubated at 26 °C until stage 45–46.

### Cyclopamine Treatment

A 5 mM stock solution of Cyclopamine (Enzo Life Sciences) was prepared in ethanol. Embryos were c by diluting the stock solution in 1/9MR + gentamycin. Control embryos were treated with a similar dilution of vehicle. Following treatment, the embryos were raised to stage 45 in 1/9 MR.

### Whole Mount Alcian Blue Staining of Cartilage

For whole mount cartilage staining stage 45 embryos were fixed in MEMFA for 20 mins at RT and then washed briefly in acid alcohol (1.2% HCL in 70% ETOH). A 0.5% alcian blue solution in acid alcohol was used to stain the embryos over night at 4 degrees. Specimens were then washed in acid alcohol several times, rehydrated into H_2_O and bleached for 2 hrs in 1.2% hydrogen peroxide under a bright light. They were then washed several times in 2% KOH and left rocking overnight in 10% glycerol in 2% KOH. Samples were processed through 20%, 40%, 60 and 80% glycerol in 2% KOH. The facial cartilages were dissected out as described previously (Griffin *et al*.^52^). A Canon EOS 5d digital camera mounted on a Zeiss discovery V8 stereomicroscope was used for imaging.

### Histology

Stage 45 embryos were fixed overnight in 4% PFA, dehydrated into MeOH, washed twice for 15 mins in Xylene, and embedded in paraffin. 10 μM sections were cut using a Leica 2295 microtome. The sections were stained by clearing the slides in Xylene (2 × 10 min.) and rehydrating them through a graded series of MeOH/H_2_O washes before placing them in 1% alcian blue 8GX (BDH) in 3% acetic acid (pH 2.5) for 10 mins. They were then washed in running water for 10 mins, rinsed briefly in H_2_O, placed into Ehrlich’s Haematoxylin (Solmedia) for 2 mins, washed in running water for 10 mins and rinsed in H_2_O. Finally, samples were dehydrated in 3 × 100% EtOH washes (2 mins each), allowed to air dry, and a cover slip placed.

### Optic Coherence Tomography

Thorlabs Telesto 1325 nm spectral domain-OCT System obtains 7 mm Imaging Depth with air-12 μm Axial Resolution (in water-9μm axial resolution). The cardiac 2D cross sectional movies obtained at high-speed mode-91 kHz (91000 A-scan per second) with 91 dB sensitivity to capture cardiac cycle, which varies between 110–140beats/min. Craniofacial images were obtained at high-sensitivity mode 5.5 kHz (5500 A-scans per second) with 106 dB sensitivity.

### MatLab – Optical Coherence Tomography interface

The task of the OCT interface is to display multiple subsequent acquired images and allow the user to measure distances between two points in the structure image or the area within a polygon. The OCT data is acquired with a Thorlabs spectral domain OCT and stored in the raw data format file. The raw file contains the unprocessed fringes from the inferometer, which has to be processed to create the final image data. The processing requires several steps and has been implemented in Matlab (Mathworks): (i) the transform matrix for the digital Fourier transform is calculated; (ii) each image is loaded and transformed column-wise into a complex-valued image; and (iii) the complex image is separated into structure and phase content. All information needed for the processing from fringe data to structural and phase image is stored in the raw data file. The axial resolution of the structure image is defined by the displacement of the light beam as defined by the user, and the refractive index of the acquired medium. Thus, the pixels in the structure image are not necessarily “squared”, meaning that the width of one pixel might be 1 μm while the height might differ from that. This has obvious implications for the measurement of distances and areas in the structure. In this work, we accommodate for this using two scaling parameters, one for each image dimension. The scaling parameters convert the distance from pixel-space to mm. When calculating a distance between two points, the pixel-distances are measured separately for each dimension, scaled by the corresponding scaling parameter and then combined into one distance in mm. Areas are calculated by measuring the area in the pixel-space and then multiplied with both scaling parameters to result in an area in mm^2^.

The graphical user interface is also created in MatLab. It consists of two main parts: (1) a panel for viewing and manipulating the structure image, and (2) a panel that shows all selected distances or areas with according measurements. The viewing panel allows the user to navigate between frames, adjust the image contrast, apply a median filter, and toggle the visibility of the selected distances and areas. All measurements are automatically saved in an accompanying file to the original raw file to be able to revisit all selected distances and areas at any time. Measurements are listed in the second panel. Each measurement is color-coded to be easily identifiable as overlay in the structure image. Measurements can be deleted or exported to clipboard to be pasted into an excel file. Two buttons allow the selection of new distances or areas. The software is available upon request.

For demonstration purposes, we converted OCT RAW data to PNG files via our MatLab interface then cropped, filtered with Gaussian Blur by Image-J (US National Institutes of Health) software prior to presentation.

### Image Acquisition

We mechanically immobilized stage 46–47 (post-fertilization Day 3) tadpoles in 2.5 ml of 1% low melt agar in a 35 × 10 mm polystyrene petri dish. 1% low melt agar was warmed to 60 °C to keep liquid but the allowed it to cool down to ~30 °C temperature before immobilizing tadpoles and then allowed to solidify at room temperature. While the agar solidified (~1 min), we manually aligned tadpoles under stereomicroscope so that ventral body and cardiac sac exposed en face to the OCT imaging field. Next, we utilized a 5-axis imaging stage (Thorlabs) which provides translation on x,y,z axis and an additional 4 degrees of freedom for yaw and tilt. Prior to full imaging by OCT, we captured a ‘reference-imaging plane’ by manually adjusting the 5-axis stage until we obtained a “symmetric mid-eye plane” (described below) which we referred to as the ‘0°- Reference - axis’. To minimize operator dependent variation, all the imaging planes are defined in respect to this reference axis.

## Additional Information

**How to cite this article:** Deniz, E. *et al*. Analysis of Craniocardiac Malformations in *Xenopus* using Optical Coherence Tomography. *Sci. Rep.* **7**, 42506; doi: 10.1038/srep42506 (2017).

**Publisher's note:** Springer Nature remains neutral with regard to jurisdictional claims in published maps and institutional affiliations.

## Supplementary Material

Supplementary Information
