# Supplementary material for: Analysis of Craniocardiac Malformations in Xenopus using Optical Coherence Tomography
Source: Sci Rep. 2017 Feb 14;7:42506. doi: 10.1038/srep42506 (PMC5307353; doi:10.1038/srep42506)
Supplement: Supplementary Information [file srep42506-s1.pdf]

**Analysis of Craniocardiac Malformations in *Xenopus* using Optical Coherence Tomography**

Engin Deniz, Stephan Jonas, Michael Hooper, John Griffin, Michael A. Choma, Mustafa K. Khokha

**Supplementary Information**

Supplementary Figure S1: **Imaging Planes and Measurements of Xenopus tropicalis heart with OCT. (a)** Tadpole heart sequential imaging from diastole to systole. **(a1)** Shows the maximal ventricle diastole where 3 separate measurements recorded from wall-to-wall. **(a2)** Shows the maximal systole where 3 separate measurements recorded from wall-to-wall. End diastolic diameter(EDD) and End systolic diameter(ESD) recorded as the average of these three measurements. **(b)** Outflow tract shown during a cardiac cycle. **(b1)** Represent the mid-systole phase where outflow tract is at the maximal stretch. **(b2 – b3)** Enlarged images from distal OFT where `distal outflow tract maximal diameter` measured as the wall-to-wall distance presented as green-a line. The `distal outflow tract maximal excursion distance` measured as the distance between the leaflets presented as yellow-b line. **(c)** Shows the tadpole heart from the lateral view during a cardiac phase. **(c1 – c2)** Focused on the proximal outflow tract during maximal diastole and systole. Inner wall-to-wall measurements made during systole presented as yellow-a line.

Supplementary Figure S2: **Cranial Imaging closely represents histology. (a/a`)** Transverse section by OCT and Histology. **(b/b`)** Coronal section by OCT and skeletal stain with Alcian Blue. **(c/c`)** Sagittal section by OCT and histology.

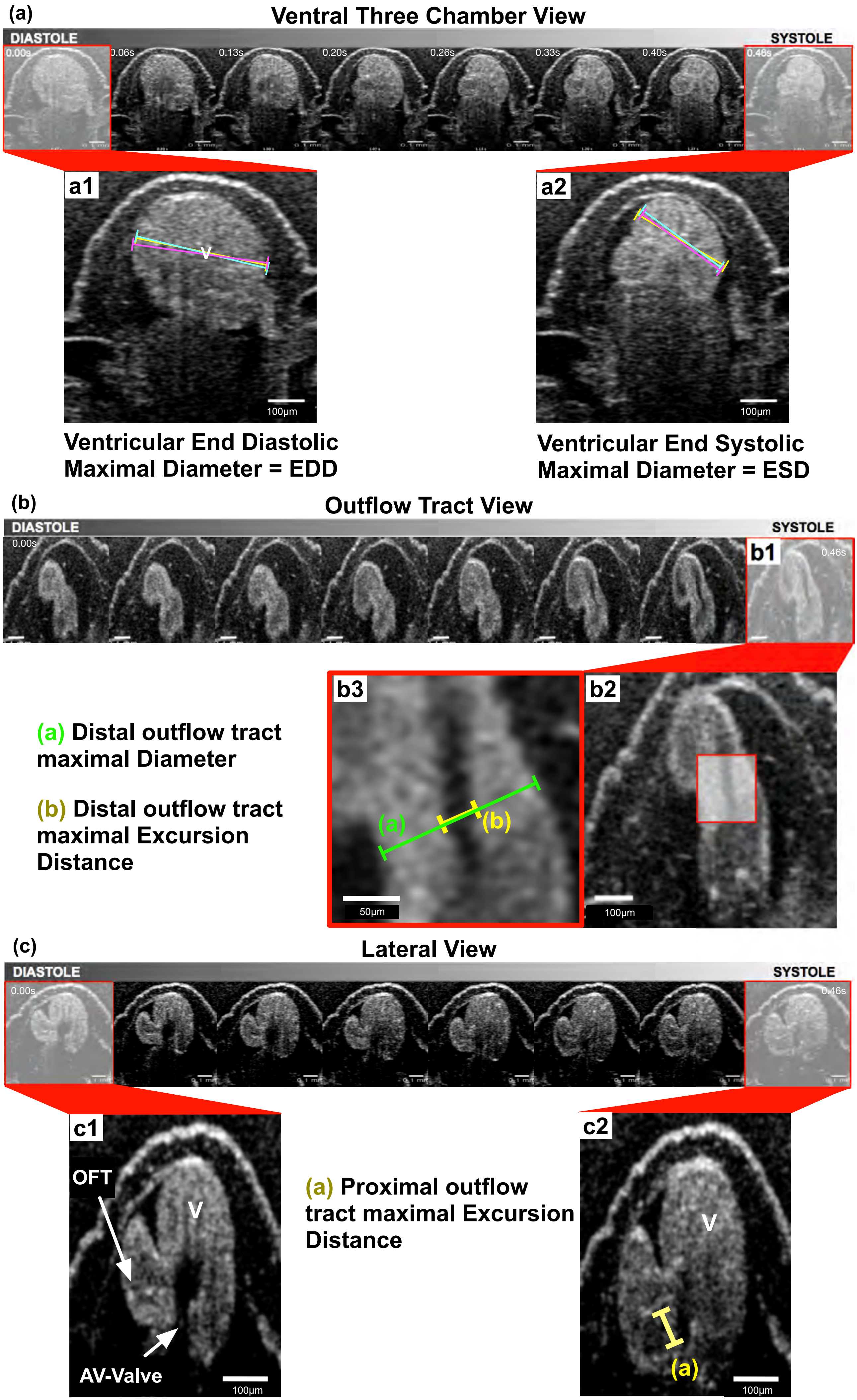
(a)
Ventral Three Chamber View
DIASTOLE
SYSTOLE
0.00s
0.06s
0.13s
0.20s
0.26s
0.33s
0.40s
0.46s
a1
100µm
a2
100µm
V
Ventricular End Diastolic Maximal Diameter = EDD
Ventricular End Systolic Maximal Diameter = ESD
(b)
Outflow Tract View
DIASTOLE
SYSTOLE
0.00s
b1
0.46s
(a) Distal outflow tract maximal Diameter
(b) Distal outflow tract maximal Excursion Distance
b3
(a)
(b)
50µm
b2
100µm
(c)
Lateral View
DIASTOLE
SYSTOLE
0.00s
0.46s
c1
OFT
V
AV-Valve
100µm
(a) Proximal outflow tract maximal Excursion Distance
c2
V
(a)
100µm

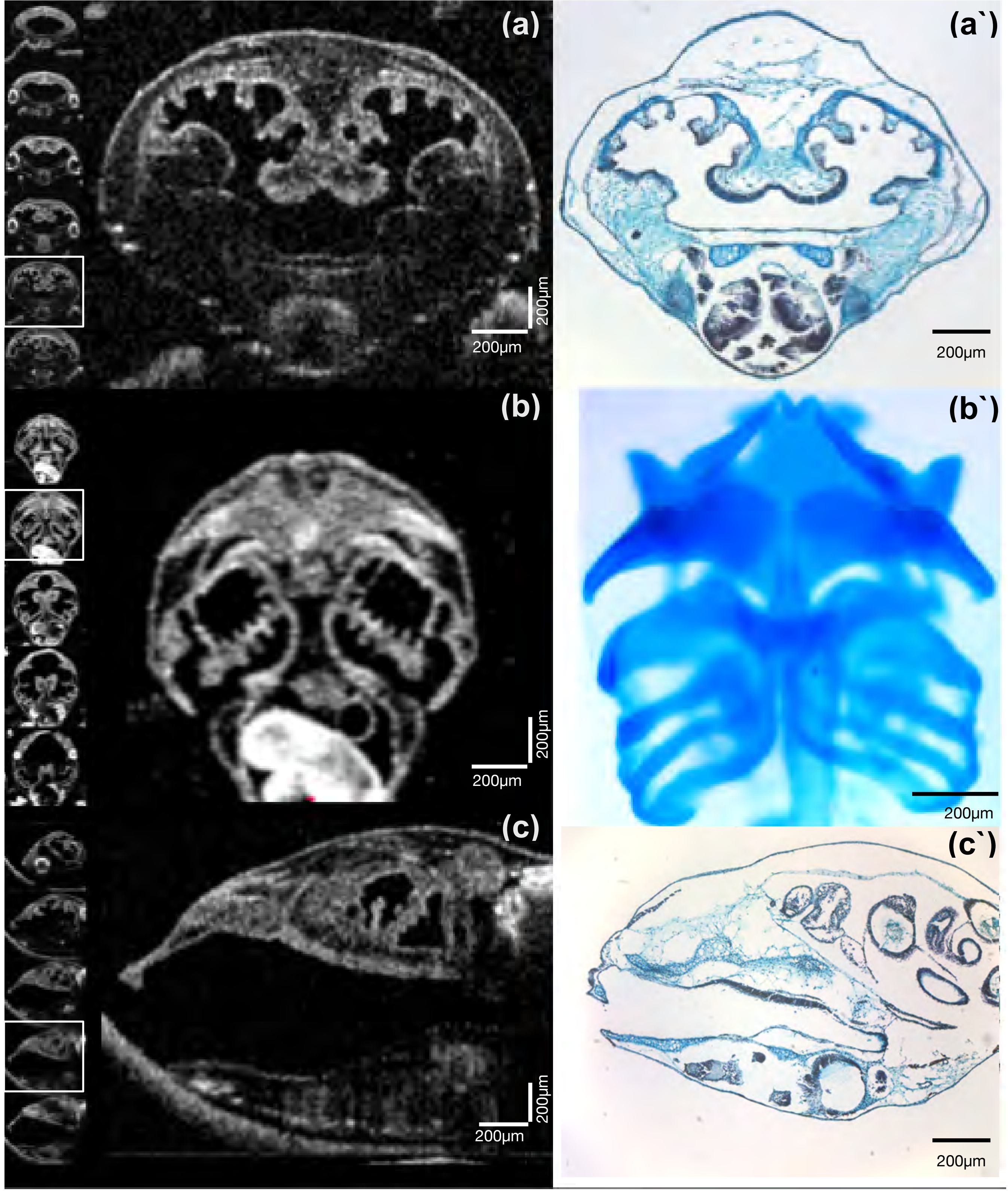

(a)
200μm
200μm
(a`)
200μm
(b)
200μm
200μm
(b`)
200μm
(c)
200μm
200μm
(c`)
200μm
